# Comparison and lessons learned from neglected tropical diseases and tuberculosis

**DOI:** 10.1371/journal.pgph.0000027

**Published:** 2021-10-13

**Authors:** Alice Wang, Adam MacNeil, Susan Maloney

**Affiliations:** Division of Global HIV & Tuberculosis, Center for Global Health, Centers for Disease Control and Prevention, Atlanta, GA, United States of America; Universidade Federal de Mato Grosso do Sul, BRAZIL

## Abstract

Currently, tuberculosis (TB) is the leading cause of death from a single infectious agent and accounts for over one-third of all HIV-related deaths. However, research and programmatic funding have lagged far behind investments for many other diseases. For about a century, the current Bacillus Calmette-Guérin vaccine has been the only effective vaccine and is only effective in preventing severe disease in children; the first new therapeutic drug for TB in over 40 years was brought to market a few years ago; and until 10 years ago, diagnosis of TB depended on a century-old testing technique. This paper relates TB to neglected tropical diseases (NTDs) and highlights shared characteristics. The aim is to elevate awareness of TB within the framework of NTDs and gain insights from successes in addressing NTDs and how these lessons can be applied to help global health programs change the trajectory of the TB epidemic. A literature review was conducted to compare TB to NTDs and highlight lessons learned from NTD control that can be applied to the TB epidemic. Common features of NTDs include underlying burden of disease, influence and effect on poverty and development, and neglect through political will and funding. There are overarching principles for the design and implementation of NTD control programs that could be applied to ending TB.

## Introduction

The World Health Organization (WHO) has identified 20 core diseases as neglected tropical diseases (NTDs), comprising primarily viral, protozoan, helminthic, and bacterial infections, as well as zoonotic or vector-borne diseases [1]. There are common features that define this group of diverse diseases [2–5]. Among the common features are that the NTDs are a proxy for poverty, often affect populations with low visibility, cause stigma and discrimination, and can be controlled, prevented, and possibly eliminated using known solutions [6]. NTDs share a heavy underlying burden of disease—both in the number of cases and the impact on the population affected, including significant economic costs for households and communities [1, 5]. Often NTDs arise in impoverished settings with poor hygiene and sanitation [3]. NTDs impair physical and cognitive development, limiting productivity and making it difficult to earn a living, thereby perpetuating a cycle of poverty and disease [3, 7]. Finally, NTDs are defined by neglect. Multiple interventions, when applied, have shown successful outcomes to decrease morbidity and mortality rates, yet funding gaps, competing priorities, and lack of political will allow NTDs to persist [2, 3].

Although tuberculosis (TB) incidence has dramatically decreased since the 18th and 19th centuries, the rise of the HIV/AIDS epidemic and drug-resistant strains of TB led the WHO to declare TB a global health emergency in 1993 [8]. Globally, an estimated 10 million people had TB disease in 2019; however, only 71% of these cases were notified to WHO, resulting in approximately 2.9 million missed TB cases (either undiagnosed or not reported [9]. Without adequate diagnosis and effective treatment, the mortality rate from TB is high [10]. In 2019, TB resulted in 1.2 million deaths among HIV-negative people and an additional 208,000 deaths among HIV-positive people [9]. Despite being curable and preventable, TB is currently the leading cause of death from a single infectious agent and accounts for over one-third of all HIV-related deaths. The goal of this paper is to elevate awareness of TB within the framework of NTDs and gain insights from successes in addressing NTDs and how these lessons can be applied to help global health programs change the trajectory of the TB epidemic.

## Underlying burden of disease

Like NTDs, TB results not only in high morbidity but also in associated economic cost, side effects of treatment, and personal stigma and isolation. Approximately 1.7 billion individuals have latent TB infection (25% of the global population) [11]. About 5%–10% of those with latent infection develop active TB during their lifetime, with differences in risk based on population. For example, the risk of TB development is much higher for children exposed to TB compared to adults, additionally for people living with untreated HIV, the annual risk of active TB disease is approximately 3%–16% [12]. Globally in 2019, 1.2 million people died of TB [9]. In addition to the lungs, TB can affect many other organ systems causing morbidity. Globally in 2018, the case fatality ratio of TB was 15%; however, if left untreated, in a 10-year time horizon, TB has a mortality rate of about 70% of patients with acid-fast bacillus (AFB) smear-positive results and 20% for those with AFB smear-negative results [13, 14].

TB and HIV co-infection can act synergistically to increase morbidity and mortality rates [15]. Patients with HIV-associated TB have an increased recurrence rate, sometimes resulting from relapse but more often from re-infection [16]. TB can be difficult to diagnose in HIV-positive patients because they may have atypical radiography, may not be able to produce a diagnostic sputum sample, often have paucibacillary disease, and are more likely to have extrapulmonary TB, which can require invasive specimen collection to confirm. Globally, around 15% of the TB disease burden is due to extrapulmonary TB [17]. However, the burden of extrapulmonary TB among HIV-positive individuals with TB is 40%–50% [17]. Additionally persons with TB and HIV co-infection must adhere to complex drug regimens that may interact with each other and potentially have overlapping toxicities [15].

A systematic review found that the total costs for TB patients in low- and middle-income countries ranged from $55 to $8198 USD, with a median 20% (range, 0%–62%) of the total cost for direct medical costs, 20% (range, 0%–84%) to direct non-medical costs, and 60% (range, 16%–94%) to income loss; half of the total cost is incurred before TB treatment initiation [18]. This systematic review also found that on average, the cost incurred was equivalent to 39% of reported household income, exceeding the threshold of 20% of the household annual income that defines catastrophic total costs due to TB [18, 19]. Diagnosis and treatment costs for multidrug-resistant (MDR) TB (resistant to isoniazid and rifampicin) and extensively drug-resistant (XDR) TB (MDR TB with additional resistance to fluoroquinolones and aminoglycosides) are considerably higher than treatment cost for drug-susceptible TB. Clearly, there is a heavy economic burden on TB patients and their families. The End TB Strategy, the WHO’s global strategy with associated targets for ending TB globally, set a milestone for zero affected families facing catastrophic costs due to TB by 2020; this milestone was not met [20].

In addition to the cost burden of TB disease, the treatment itself can be a burden. The recommended regimen for drug-sensitive TB of isoniazid and rifampicin for 6 months together with pyrazinamide and ethambutol for the first 2 months, is generally well tolerated, although the reported incidence of drug-induced liver injury, which can be caused by rifampicin, isoniazid, or pyrazinamide, varies from 5% to 33% [21, 22]. Globally, 3.3% of new TB cases and 17.7% of previously treated cases had MDR or rifampin-resistant TB [9]. Compared to drug-susceptible TB, MDR TB and XDR TB require longer and more toxic treatments. Adverse events include renal failure, hypokalemia, hypomagnesaemia, polyneuropathy, anemia, and hearing loss [10]. By the end of 2017, 127 WHO Member States have reported XDR TB cases [14]. Treatment of MDR and XDR TB is complicated, and adverse drug reactions are common. Newer drugs, shorter regimens, and all-oral MDR regimens are improving treatment outcomes, but these drugs are costly and associated with adverse effects.

In addition to the symptoms, side effects of treatment, and costs associated with TB, many patients with TB face isolation and stigma. Understanding the infectious nature of TB and associating oneself as a disease vector lead to feelings of guilt and physical and emotional isolation [23]. Many patients have reported TB-related stigma similar to that faced by HIV-positive patients [23]. In audiovisual interviews, patients with MDR and XDR TB expressed their experiences facing stigma, stating that the disease “limits you daily” and “makes your world smaller” and feeling isolated: “I am living in a box, watching the world pass me by” [10]. When looking at how TB burden has influenced disability-adjusted life-years (DALYs), the Global Burden of Disease Study in 2016 found that drug-susceptible tuberculosis resulted in 39.9 million DALYs (38.1 million to 41.9 million), multidrug-resistant tuberculosis without extensive drug resistance 3.32 million (2.79 million to 3.91 million), and extensively drug-resistant tuberculosis 369,000 (301,000–445,000) [24]. In comparison, in the same year, for the 10 NTDs defined in the London Declaration (i.e. human African trypanosomiasis, Chagas disease, Guinea worm disease, leprosy, lymphatic filariasis, onchocerciasis, schistosomiasis, soil-transmitted helminths, blinding trachoma, and visceral leishmaniasis) total DALYs were estimated at 9.0 million (5.3 million to 14.5 million) [24].

## Influence of poverty and development

Like NTDs, TB has long been defined as a disease of poverty. Globally, distinct patterns of TB disease emerge in relation to poverty and development. In 2019, of the estimated 10 million new cases of TB globally, approximately 5.4% occurred in Europe and the Americas combined, whereas Africa and South-East Asia accounted for 69% [9]. Africa has the highest TB morbidity and mortality rates, and the proportion of drug-resistant TB cases is increasing in Eastern Europe [25]. These regional disparities can be correlated with patterns of poverty and development. An ecological analysis of the incidence of TB and per capita gross domestic product (GDP) found that doubling GDP was associated with a 38.5% decrease in TB incidence [26]. However, even in high-income countries, independent of ethnicity, TB is strongly associated with poverty [27].

Poor nutrition is associated with poverty and TB disease and accelerates TB disease progression. Malnutrition can lead to secondary immunodeficiency, increasing susceptibility to TB infection [28]. The reactivation of latent TB infection may also be related to malnutrition, as chronic disease can lead to wasting in TB patients. Among tuberculin skin test positive U.S. navy recruits, TB risk was nearly four times higher among underweight men [29]. Another study in Norway found that the relative risk of TB among persons in the lowest body mass index (BMI) category was more than five times higher than the highest BMI category, independent of sex, age, and radiographic findings [30]. The link between malnutrition and TB is bi-directional. In patients with TB, reduced appetite, poor nutrient and micronutrient absorption, and altered metabolism can lead to wasting [28, 31].

Poverty is linked with substandard or inadequate housing, along with crowding and poor indoor air quality, which are all drivers for TB disease. Overcrowded housing conditions can increase TB exposure and the probability of transmission. A study across seven First Nations communities in Canada found that an increase in 0.1 persons per room was associated with a 40% increase in the risk of >2 TB cases occurring [32]. Another study looking at household contacts aged <15 years in Thailand found the risk of positive tuberculin skin testing in household contacts increased with household crowding; children living in crowded households were five times more likely to have TB infection [33]. Many studies have also implicated tobacco smoke and indoor air pollution as risk factors for TB infection, disease, and death [34]. Chronic exposure to tobacco and environmental air pollutants impairs the normal clearance of secretions on the tracheobronchial mucosal surface, which may allow *Mycobacterium tuberculosis* to bypass defense mechanisms [35].

Cultural and social barriers can trap populations disproportionately affected in a cycle of poverty and disease. TB disease is not just a product of poverty but also can lead to poverty as evidenced by the large proportion of TB patients experiencing catastrophic TB treatment costs. People in all age groups are affected by TB, but most TB cases are among working-age adults [36]. Additionally, lack of basic health services or access, poor nutrition, and inadequate living conditions contribute to TB transmission and morbidity and mortality rates. Poverty contributes to the spread of TB, and TB contributes to the persistence of poverty.

## Neglect

A huge obstacle in TB disease reduction and elimination is neglect—both in terms of funding and political will. Although funding for TB prevention, diagnosis, and treatment services has more than doubled since 2006, the Stop TB Partnership’s Global Plan to End TB 2016–2020 still estimated a gap of US$3.5 billion in TB funding in 2018 [36]. TB control funding comes from three sources: government (including loans, such as from the World Bank); the Global Fund to fight AIDS, TB, and Malaria (Global Fund); and donor agencies. Global health funding by disease highlights the neglect of TB. For TB, most funding comes from domestic sources, unlike HIV and malaria funding, therefore TB control can be a major burden for low-income countries with relatively small health budgets for competing priorities and diseases [37]. The Global Fund receives and distributes donor money from developed nations and private foundations and is the largest source of TB financing. The Global Fund disburses 18% of funding to TB and 5% to TB/HIV, compared to 29% for malaria and 48% for HIV [37]. In addition to contributions to the Global Fund, the United States government spends about 2% of its global health funding on TB, compared to 9% for malaria and 48% for HIV [38]. Additionally, the United States government funding for TB could decrease; the President’s fiscal year 2021 request proposes $283 million for TB, a decrease of $38 million from previous fiscal years, in which TB control represented only 3% of the U.S. global health budget [38].

Neglect is also evidenced by slow TB vaccine and drug development. The Bacillus Calmette-Guérin vaccine, first introduced in 1921, is still the only approved TB vaccine and only is effective at preventing severe disease in children [39]. The main anti-TB drugs (rifampicin, isoniazid, pyrazinamide, ethambutol, and streptomycin) were introduced between 1948 and 1963 [40]. Until the FDA approved bedaquiline in 2012 for MDR TB, no new anti-TB drugs with a novel mechanism of action were approved for four decades [41]. More recently in 2019, the FDA approved pretomanid for MDR TB [42]. Although other anti-TB drugs are in development, there are far fewer drug candidates for TB than for other infectious diseases, especially HIV [43]. Unfortunately, TB does not represent a substantial disease burden in high-income countries and thus is not viewed as a lucrative investment in the US, European, and Japanese pharmaceutical markets; in developing markets, new therapies are welcomed, but only if they are affordable.

Although TB targets have been set and WHO declared TB a global health emergency in 1993, lack of political will has stalled progress. In 2015, the number of TB deaths surpassed those of HIV deaths, resulting in TB as the leading global cause of infectious disease-related death. In 2015, the End TB Strategy set targets of a 90% decrease in incidence rate and 95% decrease in TB deaths (compared to 2015) by 2035 [44]. To reach these targets, the TB incidence rate needs to decrease 4%–5% annually and the mortality rates need to be 10% by 2020 [36]. However, the TB incidence rate is decreasing by only 2% annually, and though decreasing, the mortality rate is 16%, which is not enough to reach the End TB Strategy targets [36].

In 2018, heads of state gathered to discuss a concrete strategy for action and resource commitment at the United Nations High-Level Meeting (UNHLM) on TB [45]. This, in conjunction with the Multisectoral Accountability Framework, is aimed at ensuring accountability for commitments by governments and the health sector [46]. The UNHLM on TB set the target of diagnosing 40 million people with TB by 2022, including 3.5 million children and 1.5 million people with drug-resistant TB. An integral step in reaching the UNHLM TB targets is improving the accuracy and turnaround time of TB diagnostic tests. Until a decade ago, diagnosis of TB disease in most high-burden countries depended on clinical examination and a century-old testing technique, AFB smear microscopy. Although rapid TB diagnostic tests are available, most people in high-burden countries continue to be tested for TB with AFB smear microscopy, which has low sensitivity and specificity and cannot detect drug-resistant TB [10]. The advent of liquid culture media for *M*. *tuberculosis* has improved turnaround time, but, while highly accurate, can take 6 weeks for a result and requires advanced laboratories. Existing rapid diagnostics are faster, sensitive, and allow for the simultaneous detection of rifampin-resistant TB (i.e., Xpert MTB/RIF) or MDR TB (i.e., GenoType MTBDR*plus*). To reach the diagnosis commitments set by UNHLM, countries need 400% more GeneXpert modules and 600% more Xpert MTB/RIF cartridges per year—this is an incremental cost of approximately $586 million, which is 5 times more than current investments [39]. Other targets set at the UNHLM, include treating 40 million people who receive a TB diagnosis from 2018 to 2022, including 1.5 million people with drug-resistant TB, and providing TB preventive therapy (TPT) to at least 30 million people, including 4 million children and 20 million other household contacts and 6 million HIV-positive people. Although the UNHLM targets represent focused political attention on TB and hope for ending TB, these targets will require financial resources and continued political will to achieve.

### Applying NTD lessons in order to end TB

The situation for NTDs has greatly improved in the past decades, total DALYs from the London Declaration have declined 21.1% from 2010 to 2016; the success largely based on a deliberate decision to utilize population-wide preventive approaches, such as mass drug administration of preventive chemotherapy combined with broader interventions on social environmental, and economic determinants of health [24, 47]. There are overarching principles for the design and implementation of NTD control programs that could be applied to ending TB [47]. First, as both NTDs and TB affect the poor, interventions must be affordable and, if possible, made available free-of-charge. Second, interventions must be simple, safe, and undemanding since people at higher risk may live beyond the reach of effective health systems. Third, since there are few market incentives for research and development for diseases concentrated among the poor, programs should continue to move forward with what already exists in parallel with using field experience to inform ideal new interventions. Finally, community engagement is key to generate grassroots demand for treatment and reduce stigma.

In consideration of these overarching principles from NTD successes, the lens of affordability, simplicity, and grassroots demand generation should be applied to TB therapies, such as engagement of affected communities and civil society. The scale-up of therapies to treat MDR or latent TB infection provide hope for addressing TB burden. In 2019, WHO issued new guidelines for a shorter, all-oral, bedaquiline-containing regimen for the treatment of MDR TB, after programmatic data from South Africa demonstrated significantly better adherence and treatment outcomes [48]. The U.S. President’s Emergency Plan for AIDS Relief (PEPFAR) has committed to providing TB preventative therapy (TPT) to all eligible persons receiving antiretroviral therapy by 2021. In 2 years, across 16 PEPFAR countries, TPT initiations have increased 32%, and TPT completions increased 56% [49]. Additionally, through other services provided by PEPFAR and partners, several high HIV-burden countries are now on pace to control their HIV epidemic, also improving the outlook for the TB epidemic [50]. However, in contrast to increased TPT initiations and completions among people living with HIV, there is still a discrepancy in the implementation of TPT for contacts, particularly for exposed or infected children [36].

Similarly, continued demand and push for new TB diagnostics is important. New point-of-care diagnostics, scale-up of proven therapies, and epidemic control of HIV in some countries, are prospects for addressing challenges to end TB. The Xpert MTB/RIF Ultra assay (Ultra; Cepheid, Sunnyvale, CA), uses the same GeneXpert module as Xpert MTB/RIF but has improved sensitivity and a lower limit of detection of *M*. *tuberculosis*. The increased sensitivity is important for diagnosis of TB and detection of rifampin resistance, especially among patients with smear-negative results, which often is the case for HIV-positive individuals and children [51]. The recently released lateral flow urine lipoarabinomannan assays (LF-LAM) is recommended by WHO for the diagnosis and screening of TB in HIV-positive individuals with low CD4 counts or who are seriously ill [52]. Urine is easier to collect and store and lacks the infection risks associated with sputum collection. However, the sensitivity of the LF-LAM assay has yet to be optimized for use among the general population. Use of these diagnostics, especially in vulnerable populations, improves detection and early initiation of treatment.

While certain lessons learned from tackling NTDs can be applied to ending TB, it is too simplistic to suggest that TB can be ended through solely overcoming neglect, increasing funding, and boosting political will. While these factors are certainly paramount, it is important to recognize that TB vaccine development efforts have been hampered by the complexity of TB immunology and limited knowledge of clearly established correlates of protection. Additionally, civil conflicts, discrimination, and war interfere with access to care, and as long as there exist unstable political situations in which the population cannot access health care or networks of health care structures are destroyed, control of TB may not be possible. Finally, education and learning from history is important. For example, the consequence of inadequate education and mismanagement of TB patient care is a reason for the increase in MDR-TB prevalence in certain regions of the world.

## Conclusions

There are key similarities TB shares with NTDs, including the basis of its underlying burden of disease, influence and effect on poverty and development, and neglect through political will and funding. In presenting TB within the framework of NTDs, the intention is to gain insights from the successes in battling NTDs and how lessons learned can help global health programs change the trajectory of the TB epidemic, decrease TB morbidity and mortality rates and reach the End TB goals.

## References

[1] Neglected tropical diseases 2018 [Available from: http://www.who.int/neglected_diseases/diseases/en/.

[2] Molyneux DH, Hotez PJ, Fenwick A. "Rapid-impact interventions": how a policy of integrated control for Africa’s neglected tropical diseases could benefit the poor. PLoS Med. 2005;2(11):e336. doi: 10.1371/journal.pmed.0020336 16212468 PMC1253619

[3] Liese B, Rosenberg M, Schratz A. Programmes, partnerships, and governance for elimination and control of neglected tropical diseases. Lancet. 2010;375(9708):67–76. doi: 10.1016/S0140-6736(09)61749-9 20109865

[4] Hotez PJ, Molyneux DH, Fenwick A, Kumaresan J, Sachs SE, Sachs JD, et al. Control of neglected tropical diseases. N Engl J Med. 2007;357(10):1018–27. doi: 10.1056/NEJMra064142 17804846

[5] Mackey TK, Liang BA, Cuomo R, Hafen R, Brouwer KC, Lee DE. Emerging and reemerging neglected tropical diseases: a review of key characteristics, risk factors, and the policy and innovation environment. Clin Microbiol Rev. 2014;27(4):949–79. doi: 10.1128/CMR.00045-14 25278579 PMC4187634

[6] Hotez P. The neglected tropical diseases and the neglected infections of poverty: overview of their common features, global disease burden and distribution, new control tools, and prospects for disease elimination. Washington, DC: National Academies Press; 2011.

[7] Badje A, Moh R, Gabillard D, Guehi C, Kabran M, Ntakpe JB, et al. Effect of isoniazid preventive therapy on risk of death in west African, HIV-infected adults with high CD4 cell counts: long-term follow-up of the Temprano ANRS 12136 trial. Lancet Glob Health. 2017;5(11):e1080–e9. doi: 10.1016/S2214-109X(17)30372-8 29025631

[8] Tuberculosis: a global emergency. WHO report of the TB epidemic [press release]. Geneva. Report No. WHO/TB/94.177: World Health Organization 1993.

[9] Global tuberculosis report 2020. Geneva. Licence: CC BY-NCSA3.0 IGO: World Health Organization; 2020.

[10] Dheda K, Gumbo T, Maartens G, Dooley KE, McNerney R, Murray M, et al. The epidemiology, pathogenesis, transmission, diagnosis, and management of multidrug-resistant, extensively drug-resistant, and incurable tuberculosis. Lancet Respir Med. 2017. 10.1016/S2213-2600(17)30079-6 28344011

[11] Houben RM, Dodd PJ. The Global Burden of Latent Tuberculosis Infection: A Re-estimation Using Mathematical Modelling. PLoS Med. 2016;13(10):e1002152. doi: 10.1371/journal.pmed.1002152 27780211 PMC5079585

[12] Selwyn PA, Hartel D, Lewis VA, Schoenbaum EE, Vermund SH, Klein RS, et al. A prospective study of the risk of tuberculosis among intravenous drug users with human immunodeficiency virus infection. N Engl J Med. 1989;320(9):545–50. doi: 10.1056/NEJM198903023200901 2915665

[13] Tiemersma EW, van der Werf MJ, Borgdorff MW, Williams BG, Nagelkerke NJ. Natural history of tuberculosis: duration and fatality of untreated pulmonary tuberculosis in HIV negative patients: a systematic review. PLoS One. 2011;6(4):e17601. doi: 10.1371/journal.pone.0017601 21483732 PMC3070694

[14] Global tuberculosis report 2017. Geneva. Licence: CC BY-NCSA3.0 IGO: World Health Organization; 2017.

[15] Gethun H, Raviglione M, Varma JK, Cain K, Samandari T, Popovic T, et al. CDC Grand Rounds: the TB/HIV Syndemic. Morbidity and Mortality Weekly Report (MMWR). 2012;61(26):484–9. 22763886

[16] Crampin AC, Mwaungulu JN, Mwaungulu FD, Mwafulirwa DT, Munthali K, Floyd S, et al. Recurrent TB: relapse or reinfection? The effect of HIV in a general population cohort in Malawi. AIDS. 2010;24(3):417–26. doi: 10.1097/QAD.0b013e32832f51cf 20042847 PMC2917772

[17] Dheda K, Barry CE, Maartens G. Tuberculosis. Lancet. 2016;387(10024):1211–26. doi: 10.1016/S0140-6736(15)00151-8 26377143 PMC11268880

[18] Tanimura T, Jaramillo E, Weil D, Raviglione M, Lonnroth K. Financial burden for tuberculosis patients in low- and middle-income countries: a systematic review. Eur Respir J. 2014;43(6):1763–75. doi: 10.1183/09031936.00193413 24525439 PMC4040181

[19] Tuberculosis patient cost surveys: a hand book. Geneva. Licence: CC BY-NC-SA 3.0 IGO: World Health Organization; 2017.

[20] The END TB Strategy. World Health Organization; 2018 2018. Contract No.: WHO/CDS/TB/2018/29.

[21] Saukkonen JJ, Cohn DL, Jasmer RM, Schenker S, Jereb JA, Nolan CM, et al. An official ATS statement: hepatotoxicity of antituberculosis therapy. Am J Respir Crit Care Med. 2006;174(8):935–52. doi: 10.1164/rccm.200510-1666ST 17021358

[22] Treatment for TB disease Atlanta: Centers for Disease Control and Prevention; 2016 [Available from: https://www.cdc.gov/tb/topic/treatment/tbdisease.htm.

[23] Kelly P. Isolation and stigma: the experience of patients with active tuberculosis. J Community Health Nurs. 1999;16(4):233–41. doi: 10.1207/S15327655JCHN1604_3 10628114

[24] Hay S, et al. Global, regional, and national disability-adjusted life-years (DALYs) for 333 diseases and injuries and healthy life expectancy (HALE) for 195 countries and territories, 1990–2016: a systematic analysis for the Global Burden of Disease Study 2016. The Lancet. 2017;390(10100):1260–344. 10.1016/S0140-6736(17)32130-X PMC5605707 28919118

[25] Mjid M, Cherif J, Ben Salah N, Toujani S, Ouahchi Y, Zakhama H, et al. [Epidemiology of tuberculosis]. Rev Pneumol Clin. 2015;71(2–3):67–72. doi: 10.1016/j.pneumo.2014.04.002 25131367

[26] Janssens JP, Rieder HL. An ecological analysis of incidence of tuberculosis and per capita gross domestic product. Eur Respir J. 2008;32(5):1415–6. doi: 10.1183/09031936.00078708 18978146

[27] Spence DP, Hotchkiss J, Williams CS, Davies PD. Tuberculosis and poverty. BMJ. 1993;307(6907):759–61. doi: 10.1136/bmj.307.6907.759 8219945 PMC1696420

[28] Gupta KB, Gupta R, Atreja A, Verma M, Vishvkarma S. Tuberculosis and nutrition. Lung India. 2009;26(1):9–16. doi: 10.4103/0970-2113.45198 20165588 PMC2813110

[29] Edwards LB, Livesay VT, Acquaviva FA, Palmer CE. Height, weight, tuberculous infection, and tuberculous disease. Arch Environ Health. 1971;22(1):106–12. doi: 10.1080/00039896.1971.10665820 4992917

[30] Tverdal A. Body mass index and incidence of tuberculosis. Eur J Respir Dis. 1986;69(5):355–62. 3792471

[31] Macallan DC, McNurlan MA, Kurpad AV, de Souza G, Shetty PS, Calder AG, et al. Whole body protein metabolism in human pulmonary tuberculosis and undernutrition: evidence for anabolic block in tuberculosis. Clin Sci (Lond). 1998;94(3):321–31. doi: 10.1042/cs0940321 9616267

[32] Clark M, Riben P, Nowgesic E. The association of housing density, isolation and tuberculosis in Canadian First Nations communities. Int J Epidemiol. 2002;31(5):940–5. doi: 10.1093/ije/31.5.940 12435764

[33] Tornee S, Kaewkungwal J, Fungladda W, Silachamroon U, Akarasewi P, Sunakorn P. The association between environmental factors and tuberculosis infection among household contacts. Southeast Asian J Trop Med Public Health. 2005;36 Suppl 4:221–4. 16438213

[34] Lin HH, Ezzati M, Murray M. Tobacco smoke, indoor air pollution and tuberculosis: a systematic review and meta-analysis. PLoS Med. 2007;4(1):e20. doi: 10.1371/journal.pmed.0040020 17227135 PMC1769410

[35] Houtmeyers E, Gosselink R, Gayan-Ramirez G, Decramer M. Regulation of mucociliary clearance in health and disease. Eur Respir J. 1999;13(5):1177–88. doi: 10.1034/j.1399-3003.1999.13e39.x 10414423

[36] Global tuberculosis report 2019. Geneva. Licence: CC BY-NCSA3.0 IGO: World Health Organization; 2019.

[37] Fund TG. The Global Fund Data Explorer: The Global Fund; 2019 [Available from: https://data.theglobalfund.org/home.

[38] Foundation KF. Analysis of data from the Office of Management adn Budget, Agency Congressional Budget Justifications, Congressional Appropriations Bills and US Foreign Assistance Dashboard 2019 [Available from: https://www.kff.org/global-health-policy/fact-sheet/breaking-down-the-u-s-global-health-budget-by-program-area/.

[39] Tuberculosis preventive therapy in HIV-infected individuals. A joint statement of the WHO Tuberculosis Programme and the Global Programme on AIDs, and the International Union Against Tuberculosis and Lung Disease (IUATLD). Geneva: World Health Organization/International Union Against Tuberculosis and Lung Disease; 1993.

[40] Harper C. Tuberculosis, a neglected opportunity? Nat Med. 2007;13(3):309–12. doi: 10.1038/nm0307-309 17342146

[41] Mirsaeidi M. After 40 years, new medicine for combating TB. Int J Mycobacteriol. 2013;2(1):1–2. doi: 10.1016/j.ijmyco.2013.01.004 25045621 PMC4103685

[42] Keam SJ. Pretomanid: First Approval. Drugs. 2019;79(16):1797–803. doi: 10.1007/s40265-019-01207-9 31583606

[43] Working Group on New TB Drugs: Clinical Pipeline: Stop TB Partnership; 2020 [Available from: https://www.newtbdrugs.org/pipeline/clinical.

[44] Lonnroth K, Raviglione M. The WHO’s new End TB Strategy in the post-2015 era of the Sustainable Development Goals. Trans R Soc Trop Med Hyg. 2016;110(3):148–50. doi: 10.1093/trstmh/trv108 26884490 PMC4755423

[45] Herbert N, Masham BS, Suttie BA, Sharma V, Albani S, Domenti O, et al. Advancing political will to end the tuberculosis epidemic. Lancet Infect Dis. 2018;18(7):711–2. doi: 10.1016/S1473-3099(17)30679-5 29153268

[46] Multisectoral Accountability Framework to Accelerate Progress to End Tuberculosis by 2030. Geneva, Switzerland: World Health Organization; 2019.

[47] Chan M. Ten years in public health 2007–2017. The neglected tropical diseases: a rags-to-riches story. Geneva: World Health Organization; 2017. p. 79–81.

[48] Rapid Communication: Key Changes to Treatment of Drug-Resistant Tuberculosis. Geneva: World Health Organization; 2019.

[49] Melgar M, Nichols C, Cavanaugh JS, Kirking HL, Surie D, Date A, et al. Tuberculosis Preventive Treatment Scale-Up Among Antiretroviral Therapy Patients—16 Countries Supported by the U.S. President’s Emergency Plan for AIDS Relief, 2017–2019. MMWR Morb Mortal Wkly Rep. 2020;69(12):329–34. doi: 10.15585/mmwr.mm6912a3 32214084 PMC7725512

[50] Strategy for Accelerating HIV/AIDS Epidemic Control (2017–2020). In: Relief UPsEPfA, editor.: PEPFAR; 2017.

[51] WHO Meeting Report of a Technical Expert Consultation: Non-inferiority Analysis of Xpert MTF/RIF Ultra Compared to Xpert MTB/RIF. Geneva: World Health Organization; 2017.

[52] The Use of Lateral Flow Urine Lipoarabinomannan Assay (LF-LAM) for the Diagnosis and Screening of Active Tuberculosis in People Living with HIV: Policy Guidance. Geneva: World Health Organization; 2015.

